# Tuftsin-derived T-peptide prevents cellular immunosuppression and improves survival rate in septic mice

**DOI:** 10.1038/srep16725

**Published:** 2015-11-18

**Authors:** Yu-Lei Gao, Yan-Fen Chai, Ning Dong, Su Han, Xiao-Mei Zhu, Qing-Hong Zhang, Yong-Ming Yao

**Affiliations:** 1 Department of Emergency Medicine, Tianjin Medical University General Hospital, Tianjin 300052, P.R. China; 2 Trauma Research Center, First Hospital Affiliated to the Chinese PLA General Hospital, Beijing 100048, P.R. China; 3 College of Pharmacy, Nankai University, Tianjin 300071, P.R. China; 4 State Key Laboratory of Kidney Disease, the Chinese PLA General Hospital, Beijing 100853, P.R. China

## Abstract

The primary mechanisms of sepsis induced cellular immunesuppression involve immune dysfunction of T lymphocytes and negative immunoregulation of regulatory T cells (Tregs). It has been found that tuftsin is an immune modulating peptide derived from IgG in spleen. T-peptide is one of tuftsin analogs. Herein, we examined the effect of T-peptide on cell-mediated immunity in the presence of lipopolysaccharide (LPS) and the survival rate in septic mice. T-peptide regulated the proliferative ability of CD4^+^CD25^−^ T cells in dual responses. Meanwhile, 10 and 100 μg/ml T-peptides were able to enhance the apoptotic rate of CD4^+^CD25^−^ T cells compared with 1 μg/ml T-peptide, but markedly lowered interleukin (IL)-2 levels. When CD4^+^CD25^+^ Tregs were treated with T-peptide for 24 hours, and co-cultured with normal CD4^+^CD25^−^ T cells, the suppressive ability of CD4^+^CD25^+^ Tregs on CD4^+^CD25^−^ T cells was significantly lowered, along with decreased expression in forkhead/winged helix transcription factor p-3 (Foxp-3) as well as cytotoxic T lymphocyte-associated antigen (CTLA)-4, and secretion of transforming growth factor (TGF)-β. Moreover, T-peptide has the ability to improve outcome of septic mice in a dose- and time- dependent manner, and associated with improvement in the microenvironment of cellular immunosuppression in septic mice.

Sepsis is still the leading cause of death among critically ill patients in intensive care units. Furthermore, the survivors usually suffer from an impaired quality of life^1^,^2^,^3^,^4^,^5^,^6^. Administration of antibiotics against the known or experientially pathogens along with supportive care, which may help the patients survive acute stage of sepsis, frequently shift to a state of chronic morbidity lasting days to weeks^2^,^5^,^7^,^8^,^9^,^10^,^11^,^12^,^13^. There is a significant loss of immunocytes, including B/T lymphocytes, dendritic cells (DCs), gastrointestinal epithelial cells, even thymocytes from the beginning of sepsis as shown both in animal models and septic patients^10^,^11^,^14^,^15^,^16^,^17^,^18^,^19^. It has been noted that septic patients gradually enter into a state of immunosuppression after primary hyper-inflammatory response, which is defined as immunoparalysis. Therefore, it is postulated that therapeutic measures to modulate the immunosuppressive stage might be a promising interventional strategy to improve the outcome of patients.

The essential mechanisms underlying immune dysfunction of T lymphocytes include their apoptosis, a shift to anti-inflammatory cytokines, anergy as well as negative immunoregulation of Tregs and DCs, and damage of intestinal mucosa associated lymphoid tissues^6^,^7^,^8^,^10^,^11^,^13^,^14^,^15^,^16^,^17^,^18^,^19^,^20^,^21^,^22^,^23^,^24^,^25^,^26^. In recent years, investigators have become interested in studying the mechanisms with regard to immunosuppression and development of new measures to regulate immune response during sepsis, including activation of Tregs and apoptotic depletion of immunocytes. A growing body of evidence both from septic animal models and patients indicates that immunomodulatory agents, such as IL-15, IL-7, granulocyte-macrophage colony-stimulating factor (GM-CSF), anti-B/T lymphocyte attenuator (anti-BTLA), anti-programmed cell death receptor-1 (anti-PD-1), and thymosin alpha-1 may have potential ability in the treatment of septic complication^8^,^16^,^27^,^28^,^29^,^30^,^31^,^32^,^33^,^34^.

In 1970s, investigators found a natural immune modulating tetrapeptide (threonine-lysine-proline-arginine) derived from the proteolytic degradation 289–292 amino acid residues of IgG in spleen, and it was described as a phagocytosis-stimulating factor in terms of tuftsin^35^. The primary effect of tuftsin or tuftsin-like peptides was to enhance phagocyte respiratory burst, migration/chemotaxis ability, antigen presentation, and other immunologic effects of cells of monocytic origin, including macrophages, neutrophils, microglia and Kupffer cells, thereby increasing antimicrobial and antitumor activities^36^,^37^,^38^,^39^,^40^. In animal models, tuftsin or tuftsin-like peptides were found to exert therapeutic effect to improve outcome and their innate immunity. Nevertheless, the potential effect of tuftsin on adaptive immunity remains to be elucidated^38^,^39^,^40^. *In vivo*, the half life (t1/2 = 2.8 hours) of tuftsin is too short, and the structure of it is effortless decomposed by carboxypeptidase, thus it is difficult to form a drug to be used for immune-associated diseases^35^,^36^,^40^,^41^. T-peptide is one of tuftsin analogs, and it is characterized by its stable structure and long half life compared with tuftsin. The elementary structure of tuftsin-derived T-peptide is (Thr-Lys-Pro-Arg-AAN)4-(Lys-AAN)2-Lys-AAN, which has been granted a patent in China (CN-101007842A). Recently, it had been demonstrated that tuftsin-derived T-peptide was a potential postoperative adjuvant in cancer therapy, and it showed an inhibitory effect on growth of residual tumor cells after surgical resection^42^. In the current study, the objective was to investigate the effect of tuftsin-derived T-peptide on cell-mediated immunity after LPS stimulation and the survival rate in septic mice.

## Results

### The purity of CD4^+^CD25^+^Tregs and CD4^+^CD25^−^ T cells

The purity of CD4^+^CD25^+^ Tregs and CD4^+^CD25^−^ T cells was 95.93 ± 3.21% and 94.86 ± 2.89%, respectively. The viabilities of CD4^+^CD25^+^ Tregs (96.93 ± 2.29) and CD4^+^CD25^−^T cells (97.86 ± 3.48) were determined after purification using trypan blue exclusion.

### T-peptide dually regulated the proliferative activity of CD4^+^CD25^−^ T cells

**Figure 1:**
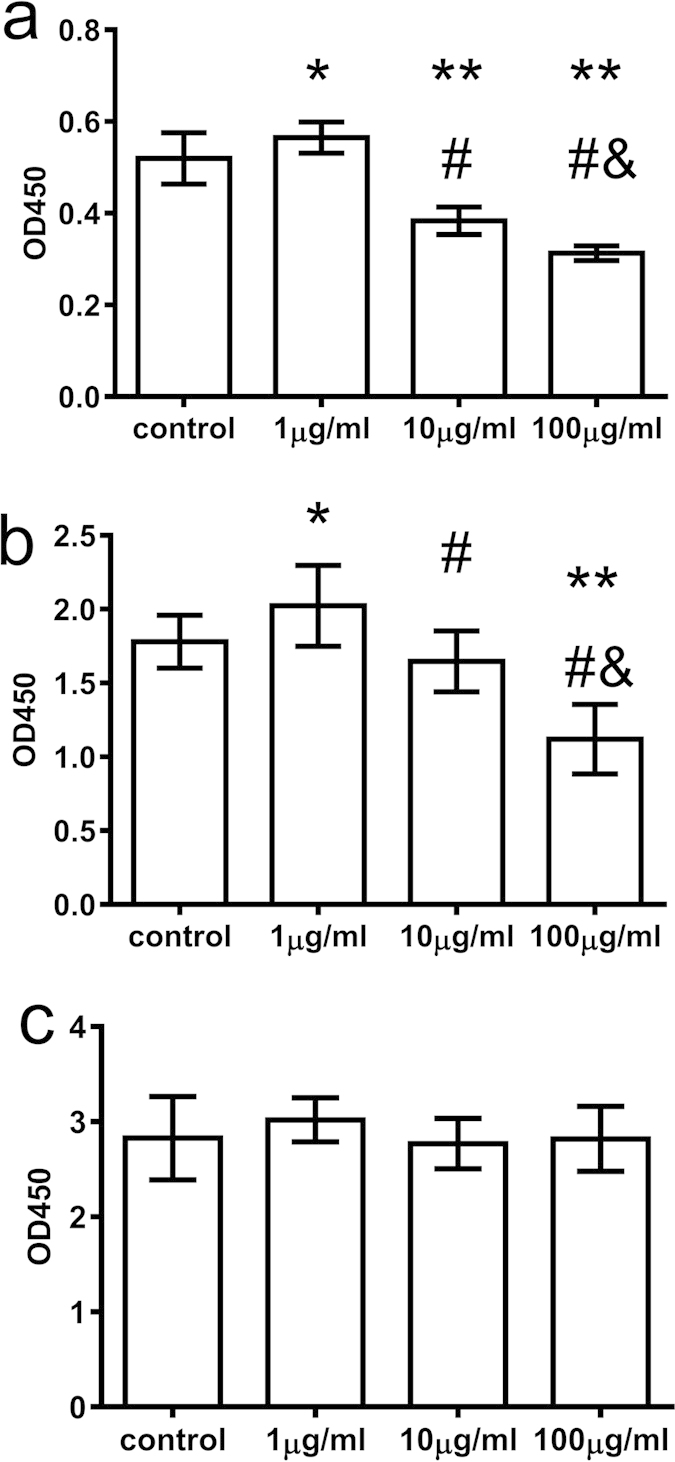
The time- and dose- dependent responses between T-peptide and the proliferative activity of splenic CD4^+^CD25^−^ T cells. The results showed the effects of various doses of T-peptide on the proliferative activity of CD4^+^CD25^−^ T cells at 12 hours **(a)**, 24 hours **(b)**, and 48 hours **(c)**, respectively. Data were represented as mean ± standard deviation (SD), and analyzed by software of SPSS 17.0 with a one-way ANOVA, n = 4 per group. Statistically significant difference as compared with the control groups, (*)*P* < 0.05, (**)*P* < 0.01; Statistically significant difference as compared with the 1 μg/ml T-peptide groups, (#)*P* < 0.01; Statistically significant difference as compared with the 10 μg/ml T-peptide groups, (&)*P* < 0.01.

As shown in Fig. 1, the proliferative rate of CD4^+^CD25^−^ T cells was enhanced from 12 (Fig. 1a) to 48 (Fig. 1c) hours with 1 μg/ml T-peptide when compared with the control group, especially at 24 hours (Fig. 1b) (*P* < 0.05). However, treatment with 10 and 100 μg/ml T-peptide significantly decreased the proliferative response of CD4^+^CD25^−^ T cells compared with the control group and the 1 μg/ml T-peptide group (*P* < 0.05 or 0.01), especially that in the 100 μg/ml T-peptide group at 24 hours (*P* < 0.05 or 0.01).

### T-peptide enhanced the apoptotic rate of CD4^+^CD25^−^ T cells

**Figure 2:**
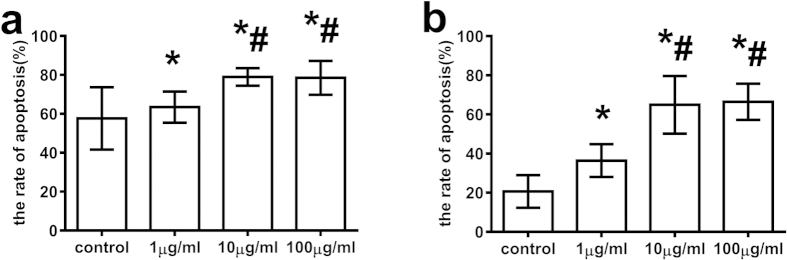
T-peptide up-regulated the apoptotic rate of CD4^+^CD25^−^ T cells. The apoptotic rate of CD4^+^CD25^−^ T cells was analyzed by annexin-V-FITC/PI flow cytometry at 12 and 24 hours after treatment. Results showed the relationship between the apoptotic rate of CD4^+^CD25^−^ T cells and different doses of T-peptide stimulation at 12 hours **(a)** and 24 hours **(b)**. Data were represented as mean ± standard deviation (SD), and analyzed by software of SPSS 17.0 with a one-way ANOVA, n = 4 per group. Statistically significant difference as compared with the control group, (*)*P* < 0.01; Statistically significant difference as compared with the 1 μg/ml T-peptide group, (#)*P* < 0.01.

Apoptotic cells were determined with the exposure of phosphatidylserine residues on the outer plasma membrane through binding of annexin-V-FITC. In parallel, dead cells were measured by PI incorporation. After stimulated for 12 and 24 hours *in vitro* (Fig. 2a,b), the apoptotic rate of CD4^+^CD25^−^ T cells in the 1 μg/ml T-peptide group was higher than that in the control group (*P* < 0.05). Treatment with 10 and 100 μg/ml T-peptide further enhanced the apoptotic rate of CD4^+^CD25^−^ T cells in comparison to the 1 μg/ml T-peptide group (*P* < 0.01), while there was no marked difference in apoptotic rate between the 10 and 100 μg/ml T-peptide groups (*P* > 0.05).

### T-peptide regulated the polarization of helper T cells and influenced IL-2 release from CD4^+^CD25^−^ T cells

**Figure 3:**
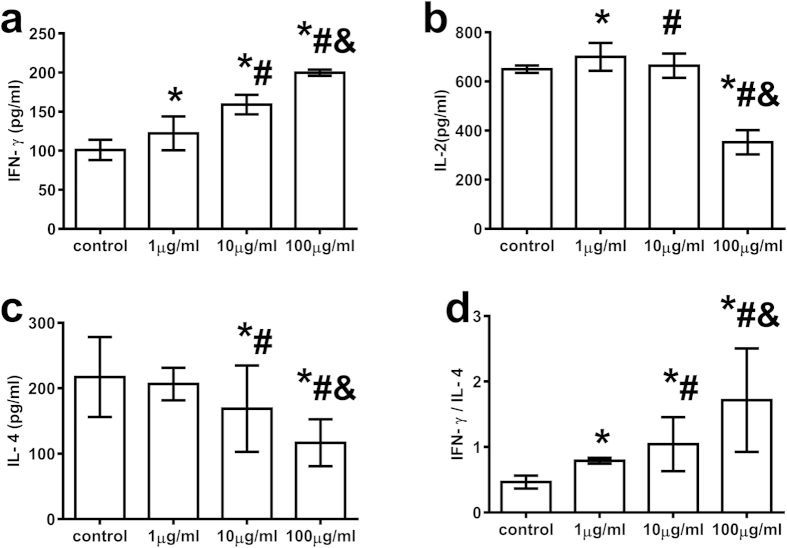
Changes in levels of secretion of IFN-γ, IL-2, and IL-4 from CD4^+^CD25^−^ T cells after 24-hour treatment with T-peptide. T-peptide could markedly enhance the release of IFN-γ **(a)**, dually regulate the release of IL-2 **(b)**, while down-regulate level of secretion of IL-4 **(c)**. T-peptide regulated the balance of IFN-γ/IL-4 of CD4^+^CD25^−^ T cells at 24 hours **(d)**. Data were represented as mean ± standard deviation (SD), and analyzed by software of SPSS 17.0 with a one-way ANOVA, n = 4 per group. Statistically significant difference as compared with the control group, (*)*P* < 0.05; Statistically significant difference as compared with the 1 μg/ml T-peptide group, (#)*P* < 0.05; Statistically significant difference as compared with the 10 μg/ml T-peptide group, (&)*P* < 0.05.

We measured IFN-γ and IL-4 levels produced by CD4^+^CD25^−^ T cells to identify polarization of helper T cells (Th)1/Th2. After treatment with T-peptide, IFN-γ level was further elevated, while IL-4 level was lowered compared with the control group in a dose- dependant manner (*P* < 0.01, Fig. 3a,c). Moreover, we investigated changes in the ratio of IFN-γ and IL-4 in order to verify the polarization of Th1/Th2 (Fig. 3d). Treatment with T-peptide markedly increased IFN-γ/IL-4 ratio compared with the control group in a dose- dependant manner (*P* < 0.05). As shown in Fig. 3b, IL-2 level was elevated after the treatment with 1 μg/ml T-peptide, while 10 μg/ml and 100 μg/ml T-peptide obviously lowered IL-2 level in comparison to the control and 1 μg/ml T-peptide groups (*P* < 0.01).

### T-peptide down-regulated the expression of Foxp-3 and CTLA-4 of CD4^+^CD25^+^ Tregs

**Figure 4:**
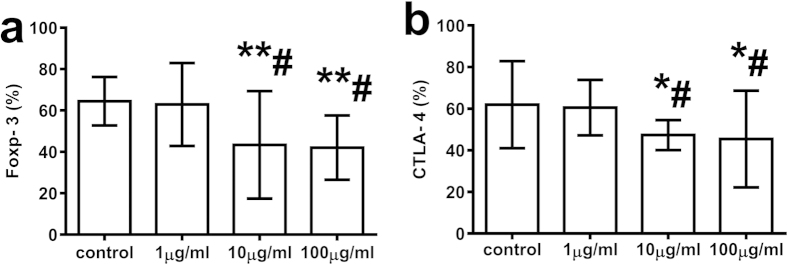
T-peptide markedly down-regulated the expressions of Foxp-3 (a) and CTLA-4 (b) of CD4^+^CD25^+^ Tregs after 24 hours. Data were represented as mean ± standard deviation (SD), and analyzed by software of SPSS 17.0 with a one-way ANOVA, n = 4 per group. Statistically significant difference as compared with the control group, (*)*P* < 0.05, (**)*P* < 0.01; Statistically significant difference as compared with the 1 μg/ml T-peptide group, (#)*P* < 0.01.

When treated with various doses of T-peptide for 24 hours *in vitro*, expressions of Foxp-3 and CTLA-4 on CD4^+^CD25^+^ Tregs were significantly down-regulated in comparison to the control group (*P* < 0.05 or 0.01). Treatment with 10 μg/ml T-peptide showed maximal response of Foxp-3 and CTLA-4 expressions compared with the 1 μg/ml group (*P* < 0.01) and the 100 μg/ml group (*P* < 0.01, Fig. 4a,b).

### T-peptide inhibited the secretion of TGF-β from CD4^+^CD25^+^ Tregs

**Figure 5:**
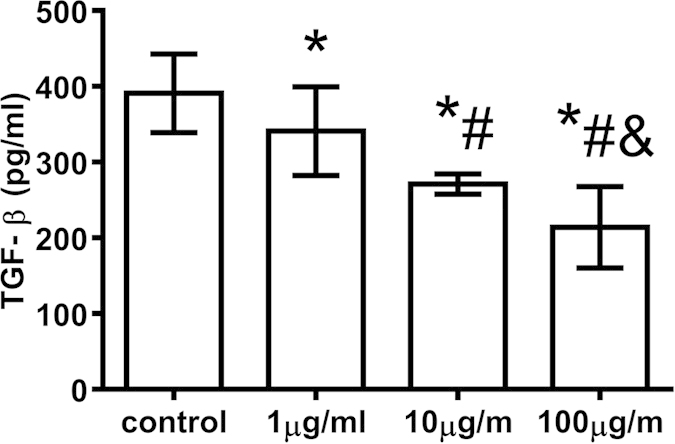
T-peptide down-regulated the secretion of TGF-β of CD4^+^CD25^+^ Tregs after 24 hours. Data were represented as mean ± standard deviation (SD), and analyzed by software of SPSS 17.0 with a one-way ANOVA, n = 4 per group. Statistically significant difference as compared with the control group, (*)*P* < 0.01; Statistically significant difference as compared with the 1 μg/ml T-peptide group, (#)*P* < 0.01; Statistically significant difference as compared with the 10 μg/ml T-peptide group, (&) *P* < 0.01.

When treated with various doses of T-peptide for 24 hours *in vitro*, the secretion of TGF-β was significantly inhibited in comparison to the control group (*P* < 0.01), especially in the 100 μg/ml group (*P* < 0.01, Fig. 5).

### T-peptide down-regulated the immunosuppressive function of CD4^+^CD25^+^ Tregs

**Figure 6:**
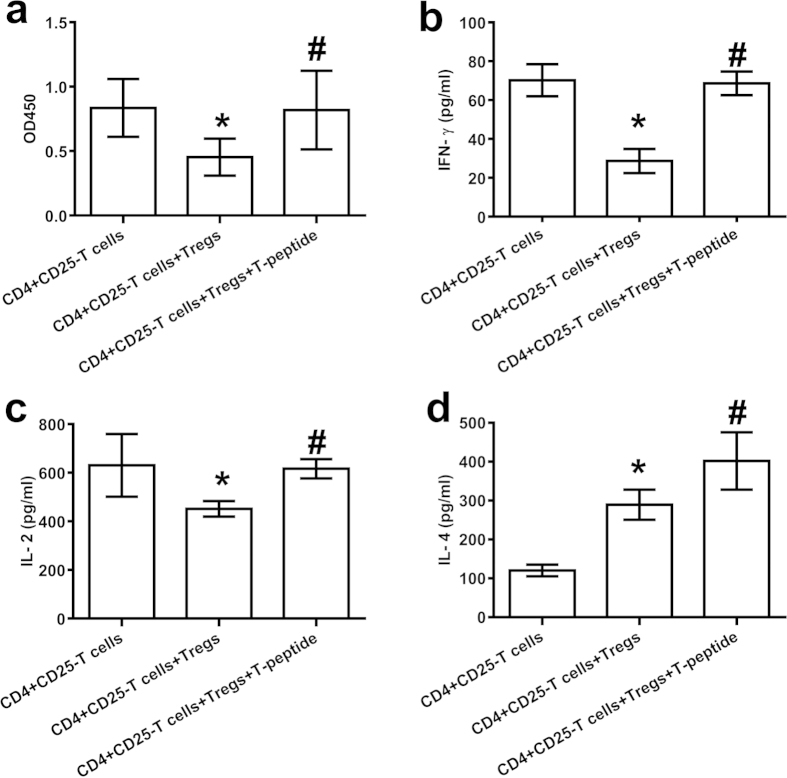
T-peptide down-regulated the immunosuppressive function of CD4^+^CD25^+^ Tregs when co-cultured with normal CD4^+^CD25^−^ T cells. After CD4^+^CD25^+^ Tregs was treated by T-peptide for 24 hours, and co-cultured with CD4^+^CD25^−^ T cells for 24 hours, the proliferative ability of CD4^+^CD25^−^ T cells (Fig. 6a), and secretion of IFN-γ (Fig. 6b), IL-2 (Fig. 6c), as well as IL-4 (Fig. 6d) of CD4^+^CD25^−^ T cells were measured. Data were represented as mean ± standard deviation (SD), and analyzed by software of SPSS 17.0 with a one-way ANOVA, n = 4 per group. Statistically significant difference as compared with normal CD4^+^CD25^−^T cell group, (*)*P* < 0.01; Statistically significant difference as compared with co-cultured without T-peptide treatment group, (#)*P* < 0.01.

In this study, we investigated the effect of T-peptide on the immunosuppressive function of CD4^+^CD25^+^ Tregs to CD4^+^CD25^−^ T cells (1:1)^43^,^44^. As shown in Fig. 6, CD4^+^CD25^+^ Tregs without T-peptide treatment significantly inhibited the proliferative activity and release of IFN-γ as well as IL-2, but enhanced the secretion of IL-4 of CD4^+^CD25^−^ T cells compared with the control group (*P* < 0.01). After CD4^+^CD25^+^ Tregs was treated by T-peptide for 24 hours, and co-cultured with CD4^+^CD25^−^ T cells, the suppressive capacity of CD4^+^CD25^+^ Tregs on CD4^+^CD25^−^ T cells was obviously diminished (Fig. 6a), and secretion of IFN-γ (Fig. 6b), IL-2 (Fig. 6c), and IL-4 (Fig. 6d) of CD4^+^CD25^−^ T cells were markedly enhanced (*P* < 0.01).

### T-peptide significantly improved the outcome of septic mice in a dose- and time- dependent manner

**Figure 7:**
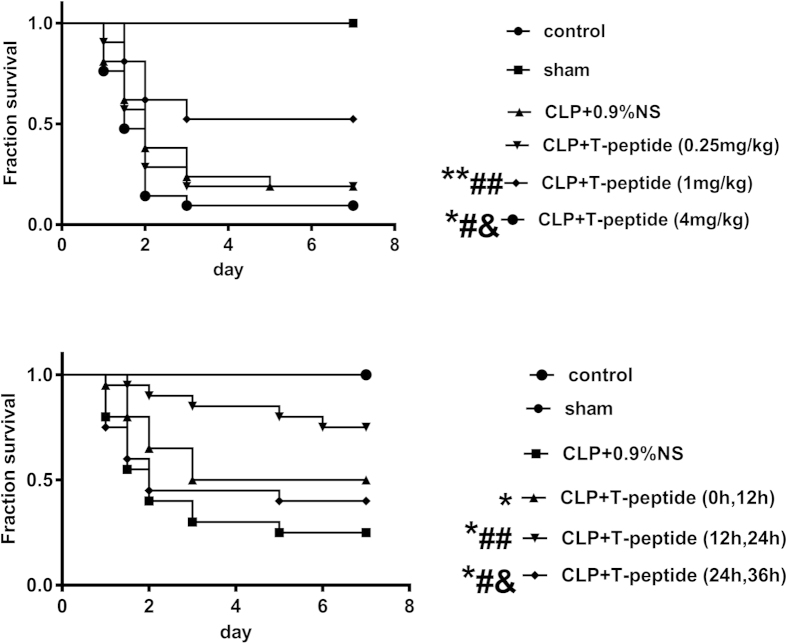
The dose- and time- dependent response between T-peptide treatment and the 7-day survival rate of septic mice. BALB/c mice were subjected to CLP to reproduce sepsis, and they received a subcutaneous injection of different doses of T-peptide (0.25, 1, and 4 mg/kg), respectively **(a)**. The survival time and rate were recorded for 7 days in various groups. Statistically significant difference as compared with CLP groups by Kaplan-Meier via the log-rank test, n = 21 per group, (*)*P* < 0.05, (**)*P* < 0.01; Statistically significant difference as compared with the CLP+ T-peptide (0.25 mg/kg) group by Kaplan-Meier via the log-rank test, n = 21 per group, (#)*P* < 0.05, (##)*P* < 0.01; Statistically significant difference as compared with the CLP+ T-peptide (1 mg/kg) group by Kaplan-Meier via the log-rank test, n = 21 per group, (&)*P* < 0.01. BALB/c mice were subjected to CLP to induce sepsis, and received a subcutaneous injection of 1 mg/kg T-peptide at various time points, respectively **(b)**. The survival time and rate were recorded for 7 days in various groups. Statistically significant difference as compared with the CLP group by Kaplan-Meier via the log-rank test, n = 20 per group, (*)*P* < 0.01; Statistically significant difference as compared with the CLP+ T-peptide (0, 12 hours) groups by Kaplan-Meier via the log-rank test, n = 20 per group, (#)*P* < 0.05, (##)*P* < 0.01; Statistically significant difference as compared with the CLP+ T-peptide (12, 24 hours) groups by Kaplan-Meier via the log-rank test, n = 20 per group, (&)*P* < 0.01.

As shown in Fig. 7a, compared with the CLP group, there was no difference in survival rate between the CLP and 0.25 mg/kg T-peptide groups (*P* > 0.05), and administration of 1 mg/kg T-peptide significantly increased the survival rate (*P* < 0.01). However, 4 mg/kg T-peptide obviously decreased survival rate (*P* < 0.01). We used 1 mg/kg T-peptide to further observe the time-dependent effect of T-peptide on survival rate of septic mice following CLP. As shown in Fig. 7b, compared with the CLP group, the survival rate of septic mice was improved in various extent at three time points in the 1 mg/kg T-peptide groups (*P* < 0.01). First injection of T-peptide at 12 hours after CLP was shown to be best time compared with other two time points (*P* < 0.05 or 0.01). Therefore, treatment with 1 mg/kg T-peptide at 12 hours after CLP was the most appropriate time.

### Treatment with T-peptide significantly improved the dysfunction of cellular immunity in septic mice

**Figure 8:**
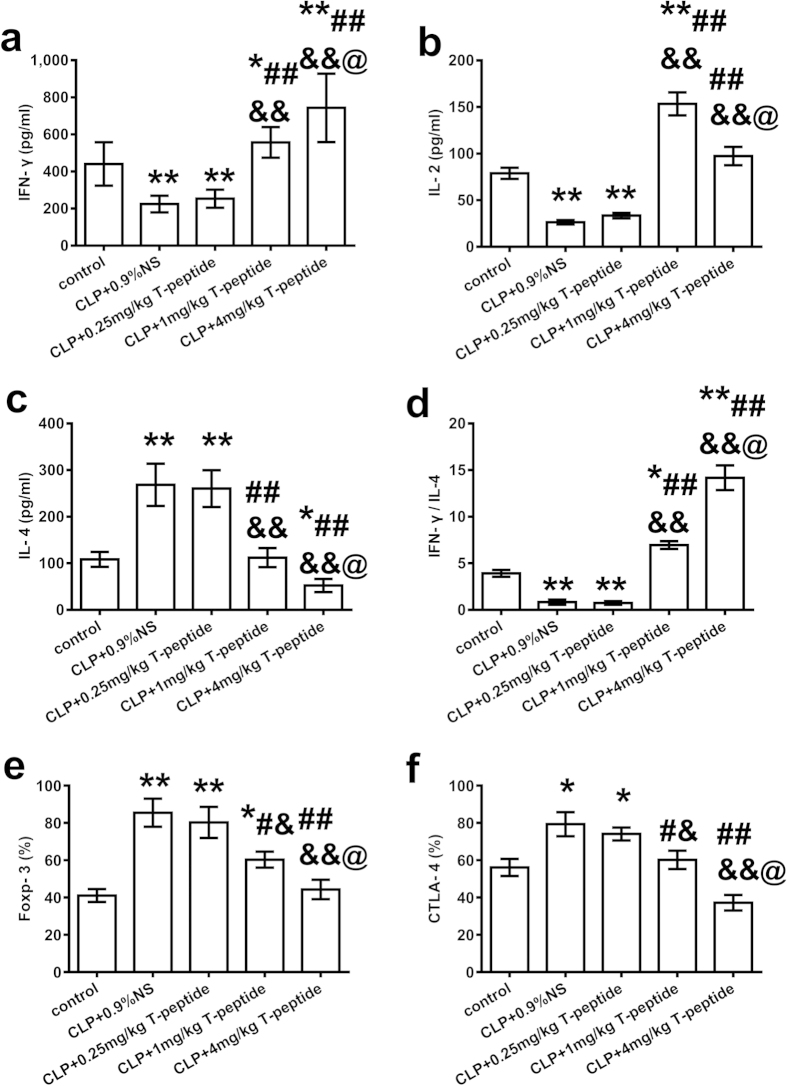
Changes in serum levels of IFN-γ, IL-2, and IL-4, as well as expressions of Foxp-3 and CTLA-4 on splenic CD4^+^CD25^+^ Tregs at 24 hours after treatment with T-peptide in septic mice. Treatment with T-peptide could markedly elevate serum level of IFN-γ **(a)**, dually regulate IL-2 level **(b)**, while down-regulate the level of IL-4 **(c)**, and modulate the balance of IFN-γ/IL-4 **(d)** at 24 hours in a dose-dependent manner. The expressions of Foxp-3**(e)** and CTLA-4**(f)** on splenic CD4^+^CD25^+^ Tregs were down-regulated at 24 hours after treatment with T-peptide in septic mice. Data were represented as mean ± standard deviation (SD), and analyzed by software of SPSS 17.0 with a one-way ANOVA, n = 4 per group. Statistically significant difference as compared with the control group, (*)*P* < 0.05, (**)*P* < 0.01; Statistically significant difference as compared with the CLP + 0.9% NS group, (#)*P* < 0.05, (##)*P* < 0.05; Statistically significant difference as compared with the CLP + 0.25 mg/kg T-peptide group, (&)*P* < 0.05, (&&)*P* < 0.05; Statistically significant difference as compared with the CLP +1 mg/kg T-peptide group, (@)*P* < 0.05.

We investigated the effect of T-peptide on the dysfunction of cellular immunity in septic mice, including serum levels of IFN-γ, IL-2, and IL-4, as well as expressions of Foxp-3 and CTLA-4 on splenic CD4^+^CD25^+^ Tregs at 24 hours after treatment with T-peptide in septic mice. As shown in Fig. 8, compared with the CLP group, there were no marked differences in serum levels of IFN-γ, IL-2, and IL-4, as well as expressions of Foxp-3 and CTLA-4 on splenic CD4^+^CD25^+^ Tregs between CLP and 0.25 mg/kg T-peptide groups (*P* > 0.05). Administration of 1 mg/kg and 4 mg/kg T-peptide significantly elevated serum level of IFN-γ, while IL-4 level was significantly lowered compared with CLP and 0.25 mg/kg T-peptide groups in a dose-dependant manner (*P* < 0.01, Fig. 8a,c). Moreover, treatment with T-peptide significantly increased IFN-γ/IL-4 ratio compared with the CLP group in a dose-dependant manner (*P* < 0.01, Fig. 8d). IL-2 level was obviously elevated after treatment with 1 mg/kg and 4 mg/kg T-peptides compared with the CLP group (*P* < 0.01, Fig. 8b), but 4 mg/kg T-peptide was found to decrease IL-2 level in comparison to 1 mg/kg T-peptide group (*P* < 0.05). In the T-peptide treated groups, expressions of Foxp-3 and CTLA-4 on CD4^+^CD25^+^ Tregs were markedly down-regulated in comparison to the CLP group, showing in a dose-dependant pattern (*P* < 0.05 or 0.01, Fig. 8e,f).

## Discussion

In the current study, we first reported that tuftsin-derived T-peptide had a marked therapeutic effect on improvement of the outcome of severe sepsis in a dose- and time-dependent manner via regulating host immune response, especially T cell-mediated immunity. It has been known that immune dysfunction of CD4^+^ T lymphocytes is one of the primary cellular mechanisms in sepsis-induced immunosuppressive state. Immediate observation of specimens of spleen, thymus, and lung in septic patients who died in intensive care units or murine CLP model showed a profound, progressive, apoptosis-induced loss of adaptive immunocytes, thereby resulting in a decrease in ability of producing antibodies and clearing life-threatening pathogens^7^,^10^,^11^,^15^,^17^,^18^,^19^. It is well known that the activated CD4^+^ T cells would differentiate into mainly T helper cells (Th)1 and Th2, which mainly produced IFN-γ and IL-4, respectively^12^. A shift to Th2 response was corroborated in sepsis-induced immune suppression, and the secretion of Th1 associated cytokines was thereby impaired, and on the other hand the secretion of Th2 associated cytokines was increased during sepsis, and this phenomenon was obviously correlated with the outcome of septic complications^11^,^24^. In the current study, it was noticed that administration of T-peptide enhanced the secretion of Th1 associated cytokine (IFN-γ), while the secretion of Th2 associated cytokine (IL-4) level was significantly lowered, and the response of cell-mediated immunity shifted to Th1. Nevertheless, hypernomic administration of T-peptide caused hyper-inflammatory response, thereby leading to the damage of organs, which might be related to the decreased survival rate of septic mice after treatment with 4 mg/kg T-peptide. T cells are anergic when they respond to their specific antigens, which are identified as failure to proliferate or secrete cytokines. IL-2 is mainly produced by CD4^+^ T lymphocytes, and it is used to support proliferation of antigen-stimulated T cells. It is simultaneously conducive to maintain the dynamic equilibrium of lymphocytes by activating induced cell death^45^. The importance of IL-2 in cleaning life-threatening pathogens has been demonstrated in septic model using knockout mice^46^. Therefore, preventing T cell apoptosis, promoting T cell proliferation, preserving balance of Th1/Th2 response, and enhancing Th1 response would be conductive to self-preservation secondary to septic challenge^6^,^10^,^11^,^12^.

In the present study, we showed that T-peptide had the ability to regulate immune dysfunction of CD4^+^ T lymphocytes *in vitro*. 1 μg/ml T-peptide could enhance the proliferative activity of CD4^+^CD25^−^ T cells, but 10 and 100 μg/ml T-peptide significantly down-regulate the proliferative response from 12 to 48 hours. We further investigated the apoptosis of CD4^+^CD25^−^ T cells and IL-2 formation, and noticed that the proliferation of CD4^+^CD25^−^ T cells was correlated with its apoptotic rate or IL-2 release. T-peptide with dose higher than 10 μg/ml possessed ability to increase the apoptotic rate of CD4^+^CD25^−^ T cells together with significant lowering of IL-2 formation, while 1 μg/ml T-peptide obviously elevated IL-2 level. Both proliferation and apoptosis of CD4^+^ T lymphocytes were concomitantly observed in experimental septic models and septic patients^6^,^11^,^18^. In patients, at the early stage of severe sepsis, proliferation/activation and apoptosis of CD4^+^ T lymphocytes were found at the same time^18^. At present, proliferation and apoptosis of CD4^+^ T lymphocytes and the relationship between them were not systematically elaborated in the setting of sepsis. Roger and his colleagues showed that inhibition of T cell apoptosis and exaggeration of T cell proliferation appeared to be associated with a higher amount of IL-2 secretion. *In vitro* study, they observed a marked increase in IL-2 release in hematological CD4^+^ T lymphocytes of septic patients after overnight costimulation with CD3/CD28, and there was a positive correlation to CD4^+^ T lymphocyte proliferation, which was in accordance with the findings in septic mice model^18^,^46^. Therefore, it is our belief that T-peptide might regulate proliferation and apoptosis of CD4^+^ T lymphocytes at least in part via IL-2 formation. On the other hand, it was of importance that T-peptide markedly shifted the polarization of helper T cells in CD4^+^CD25^−^ T cells. After administration of T-peptide, Th1 response was increased, while Th2 response was diminished. The balance of Th1/Th2 was gradually reversed to normal range, contributing to the improvement of host immune response as a result of septic episode.

Tregs, which are involved in sepsis-induced immunosuppression, are characterized by the expression of Foxp3 and CD25 (IL-2Rα-chain), and play essential roles in maintaining immune homeostasis, preventing autoimmunity, and modulating inflammatory response to infection^7^,^13^,^34^,^47^. Foxp-3 is a distinctive transcriptional factor of Tregs, and it is critical for their function, differentiation, and maintenance. Our previous study indicated that a significantly increased expression of Foxp-3 in Tregs was positively correlated to the mortality of burn-induced septic mice^26^. In the development of sepsis, Tregs can mainly inhibit the activation of T lymphocytes, especially CD4^+^ T lymphocytes through various suppressive mechanisms, including suppression by inhibitory cytokines and cell-to-cell contact (such as CTLA-4 and membrane associated TGF-β), inhibition of cytolysis (especially granzymes), down-regulation of metabolic disruption, and suppression of maturation and function of antigen presenting cells^15^,^16^,^21^,^26^. Accumulative evidence has shown that a combination of Foxp-3, CTLA-4, membrane-associated TGF-β, and inhibitory cytokines (IL-10 and TGF-β) might serve as active markers for Tregs in sepsis, and it appeared to be involved in the immunosuppressive ability of Tregs on CD4^+^ T lymphocytes, including apoptosis, a shift to anti-inflammatory cytokines, and anergy^43^,^44^. In the current study, it was noted that T-peptide could down-regulate the suppressive activity of CD4^+^CD25^+^ Tregs, and the expression of Foxp-3 and CTLA-4 as well as the secretion of TGF-β were significantly lowered by treatment with immune stimulatory agent. In addition, treatment with T-peptide diminished the suppressive ability of CD4^+^CD25^+^ Tregs on CD4^+^CD25^−^ T cells, and the proliferative activity as well as IL-2 formation of CD4^+^CD25^−^ T cells were obviously increased together with reversed balance of Th1/Th2 response. These findings were in accordance with the results of our previous studies that down-regulation of activity of CD4^+^CD25^+^ Tregs through *Astragalus Polysaccharides* or high mobility group box-1 protein marked enhanced cell-mediated immunity by modulating the proliferation of CD4^+^CD25^−^ T cells and the polarization of helper T cells, which was associated with improvement in outcome of burn-induced septic mice and decreasing the probability of secondary infection with *P. aeruginosa*^26^,^43^,^44^.

Recent studies have shown that neuropilin-1 (Nrp-1) is identified as a receptor for tuftsin on microglial cells and endothelial cells^38^,^41^. Nrp-1 is characterized as a single-pass transmembrance glycoprotein originally described to be involved in anon guidance, angiogenesis and timorous growth, and served as a receptor for the class 3 semas and members of vascular endothelial growth factor family^37^,^41^. More recently, Nrp-1, an essential component of the immunological response in humans and animals, is identified as a potent surface maker in Tregs^48^,^49^. Thus, we hypothesized that the mechanism of T-peptide on Tregs might be mediated by Nrp-1.

## Materials and Methods

### Animals

Inbred male BALB/c mice (Laboratory Animal Center of Chinese Academy of Medical Sciences, Beijing, China), 6–8 weeks old, weighing 20 ± 2 g, were used in the present study. The permit number of animal is SCXK-Jing-2009-0007. All procedures were undertaken in accordance with the National Institute of Health Guide for the Care and Use of Laboratory Animal, and approved by the Scientific Investigation Board, Medical College of Chinese PLA, Beijing, China.

### Medium and reagents

The purity of tuftsin-derived T-peptide was 99.5%. Dry preparation of tuftsin-derived T-peptide was stored at −20 °C, and it was dissolved to different concentrations in RPMI1640 with 10% fetal calf serum (FCS) or 0.9% sterile saline solution before use. The medium used throughout the *in vitro* experiment was RPMI1640 (containing 100 U/ml penicillin, 100 μl/ml streptomycin and 1.5 mM glutamine) with 10% heat-inactivated FCS. CD4^+^CD25^+^ regulatory T cell isolation kits [containing 1 ml cocktail of biotin-conjugated monoclonal anti-mouse CD8a, CD11b, CD45R, CD49b and Ter-119, 2 ml anti-biotin microbeads, 1 ml phycoeryanate (PE)-conjugated mouse CD25 and 1 ml anti-PE microbeads]. LD/SM columns were purchased from Miltenyi Biotec GmbH, Bergisch Gladbach, Germany. Cell counting kit-8 (CCK-8) was used to determine the proliferative activity of CD4^+^CD25^−^ T cells, and it was purchased from Dojindo, Kumamoto, Japan. Annexin V-fluorescein isothiocyanate (FITC) apoptosis kit [containing 50 ml binding buffer, 500 μl annexinV-FITC and 250 μl propidium iodide (PI)] was purchased from Nanjing Keygen Biotech, Nanjing, China. LPS from *Escherichia coli* 0111:B4 was purchased from Sigma, St. Louis, MO. Purified hamster anti-mouse CD3e and CD28 were purchased from BD Pharmingen, San Diego, CA. Antibodies used for flow cytometric analysis, including FITC-conjugated anti-mouse/rat- Foxp-3, FITC-conjugated anti-mouse CD152 (CTLA-4), FITC-conjugated anti-mouse/rat-CD4 and PE-conjugated anti-mouse/rate-CD25, were purchased from eBioscience, San Diego, CA. Enzyme-linked immunosorbent assay (ELISA) kits for interferon (IFN)-γ, IL-2, IL-4, and TGF-β were purchased from Excell Biol, Shanghai, China. Ketamine and Su-Mianxin-II (containing 2,4-xylazole, ethylenediaminetetraacetic acid, dihydroetopine and haloperidol) were purchased from China Academy of Military Medical Sciences, Beijing, China, and they were used as anesthesia for animals.

### Isolation of splenic CD4^+^CD25^+^ Tregs and CD4^+^CD25^−^ T cells

Normal mice were sacrificed and spleens were harvested. Mononuclear cells were obtained from passing spleens through a 30 μm stainless steel mesh twice, and then treated with Ficoll-Paque density gradient centrifugation. CD4^+^CD25^+^ Tregs and CD4^+^CD25^−^ T cells were isolated from mononuclear cells using mouse CD4^+^CD25^+^ regulatory T cell isolation kit and MiniMACS^TM^ separator (Miltenyi Biotec GmbH, Bergisch Gladbach, Germany) according to manufacturer’s instructions.

### Sepsis model

After being anesthetized, a 0.5 cm incision was made on the abdomen of mice, and the cecum was exposed. The cecum was ligated at half the distance between its distal pole and ileocecal junction, and a single puncture was made through the cecum. The diameter of needle was 0.6 mm, which was used to induce CLP customarily in such experiment. The abdominal incision was closed using simple running sutures. The mice were given subcutaneous injection of 0.9% sterile saline solution in 40 ml/kg body weight after CLP.

### Experimental design

*In vitro* study, CD4^+^CD25^+^ Tregs and CD4^+^CD25^−^ T cells were subsequently seeded on 96-well (2 × 10^5^/well) cell culture plates, and they were treated with anti-CD3 (5 μg/ml) and anti-CD28 (2 μg/ml) antibody for polyclonal activation of T cells, respectively. Cells were then stimulated with T-peptide for different intervals of 12, 24 and 48 hours or in different doses of 1, 10 and 100 μg/ml with LPS (100 ng/ml). CD4^+^CD25^+^ Tregs were stimulated for 24 hours by T-peptide, and then they were co-cultured with normal CD4^+^CD25^−^ T cells were for 24 hours. After being stimulated, the proliferative activity, apoptotic rate as well as secretion ability (including IFN-γ, IL-2 and IL-4) of CD4^+^CD25^−^ T cells, and the expression of Foxp-3/CTLA-4 of CD4^+^CD25^+^ Tregs were determined.

*In vivo* study, 126 mice were used to investigate the dose-dependent effects of T-peptide on the survival rate of severe sepsis, and they were divided into six groups: control group, sham group, CLP group, and CLP with three different doses of T-peptide treatment groups (0.25, 1, and 4 mg/kg), 21 mice in each group. The first administration of T-peptide was immediately after CLP, and T-peptide was given again at 12 hours. Another 120 mice were employed to observe the time-dependent effects, and they were divided into six groups: control group, sham group, CLP group, and CLP with three different time points of T-peptide treatment groups, 20 mice in each group. In the CLP with T-peptide treatment groups, the first administration time point of T-peptide respectively was 0, 12 and 24 hours after CLP, and T-peptide was given again 12 hours later. Sham and CLP groups were treated with equal volume of 0.9% sterile saline solution. The survival time and rate were recorded for 7 days in various groups.

### CCK-8 measurement

The proliferative activity of CD4^+^CD25^−^ T cells was determined by CCK-8 according to protocols provided by the manufacturer. The absorbance was read in microplate reader (Spectra MR, Dynex, Richfield, MN) at OD450 nm.

### Flow cytometric analysis

CD4^+^CD25^+^ Tregs were stained with FITC-conjugated anti-mouse-CTLA-4 for 30 minutes at 4 °C in the dark. For determination of intranuclear Foxp-3, CD4^+^CD25^+^ Tregs were suspended in 1 ml fixation/permeabilization solution for 2 hours at 4 °C in the dark. After washing cells with 1× permeabilization buffer twice, CD4^+^CD25^+^ Tregs were stained with FITC-conjugated anti-mouse/rat-Foxp3 for 30 minutes at 4 °C in the dark. After washing CD4^+^CD25^+^ Tregs with PBS twice, cells were analyzed by flow cytometer (BD Biosciences, Mountain View, CA) after the following procedures. 5 × 10^5^ − 1 × 10^6^ CD4^+^CD25^−^ T cells were washed in PBS twice, cells were suspended in 200 μl 1× binding buffer, followed by 10 μl FITC-conjugated annexin-V to stain for 30 minutes at 4 °C or 15 minutes at 25 °C in the dark. 300 μl 1×binding buffer and 5 μl PI were added to stain for 5 minutes at 25 °C in the dark again, and they were subjected to flow cytometric analysis by flow cytometer.

### ELISA measurement

The supernatants were collected for measurement of IFN-γ, IL-2, IL-4 and TGF-β levels by ELISA kits, strictly according to the protocols provided by manufacturer. 100 μl of ortho-phosphoric acid was added to terminate the color reaction. Plates were read in microplate reader at OD 450. The standard concentration curve for IFN-γ, IL-2, IL-4 and TGF-β were plotted from 0 to 1000 pg/ml. Examination of all samples was run in quintuplicates.

### Statistical analysis

Data were represented as mean ± standard deviation (SD), and analyzed by software of SPSS 17.0 with a one-way ANOVA. Unpaired Student’s *t*-test was used to evaluate significant differences between groups. A *P*-value of 0.05 or 0.01 was considered statistically significant. Survival rate in septic mice was evaluated by Kaplan-Meier via the log-rank test.

## Additional Information

**How to cite this article**: Gao, Y.-L. *et al.* Tuftsin-derived T-peptide prevents cellular immunosuppression and improves survival rate in septic mice. *Sci. Rep.* **5**, 16725; doi: 10.1038/srep16725 (2015).
